# New Medical Schools

**Published:** 1848

**Authors:** 


### ARTICLE I.

#### NEW MEDICAL SCHOOLS.

Two new medical schools have been advertised to go into operation the present fall in the North-West; one at Rock Island, Illinois, and the other at Iowa city, Iowa; and we hear that it is proposed soon to establish one at Indianapolis, Ind., and one at Ann Arbor, Michigan. Neither of these points presents the advantages of a Hospital in which might be given clinical instruction; nor is it probable that any of them save, perhaps, Indianapolis, can have facilities for prosecuting the study of practical Anatomy, sufficient to justify a dissecting class of twenty-five students.

Then, in reference to the demand for them by the profession, it certainly cannot be found far beyond the learned few who enter into the faculties, and we think a few years' experience will teach them that the demand that is thus created upon their time and means will more than compensate for any urgency in the necessity for the schools.

The history of all efforts to sustain medical schools in small towns, shows that it is done, if done at all, at great expense and by the most labored exertions. And even when with these they attain to any degree of prosperity, it is quite transient. Look at the school at Fairfield, New York. Although having a strong faculty, the disadvantages of the site were such as to cause its suspension. Look at the Willoughby Medical School in Ohio. It struggled on through many years and finally removed to a larger town. We might enumerate many others.

Nor is this all. Men of good abilities are not much disposed to continue a lame institution, even in a large town.

And every school without clinical instruction must be regarded as lame. Look at the medical department of the Cincinnati College. It flourished for a number of years, while the Marine Hospital of Cincinnati was under the care of the faculty. But with its largest class at its last session, numbering over two hundred students, and as strong a faculty as has been organized in the West, being composed of such men as Drake, Gross, Parker, Harrison, Rogers, McDowell, and Rives, it was suspended because the privilege of teaching in the Hospital was denied the faculty.

Although we do not believe the large classes that attend some of our eastern medical schools are advantageous, because rendering the opportunities of witnessing the demonstrations bad, still no medical school should be started where, from the patronage it would naturally command with an able faculty, its classes must be kept below two hundred paying students. The prospect of this amount of patronage, will be necessary to induce men of ability to engage in so expensive an enterprise—it will be necessary to justify the expenditures that are incident upon fitting up an institution properly—it will be necessary to justify the proper amount of labor and pains upon the course by the members of the faculty.

But there is another and more important consideration—medical schools in small towns are, of course, without the important—the indispensable—advantanges of hospital teaching and a supply of the means of practical anatomical demonstration, and they are under the necessity of offering some other inducements to call students to their classes. These are found almost universally in low charges and unlimited credit. In fact, without these inducements, few would attend such institutions for the reason that if they have to pay they will go where the advantages are better.

An instance in hand is not far distant where it was thought necessary by a Medical School to accept an amendment of its charter, which allows two students from each county in the State in which it is located, (186 students), to take out the tickets at half price, for which no compensation was offered save the preamble, "for the promotion of the cause of Education," being prefixed to the amendment. The amendment was evidently solicited by the schools to give its faculty a cloak under which to reduce their fees to half price, which makes them less than $5 for each Professor's ticket.

Look at the map of the North-West, and you will find that there are large cities enough, and at convenient distances, to afford all the facilities that can possibly be demanded by the profession for medical colleges. Then where the necessity of starting new schools in small towns?

The common opinion of the medical profession of the United States, as expressed through the National Medical Association, being adverse to the pretensions of such schools, cannot have other than a good effect in preventing enterprises of this kind when its influence shall be generally felt. E.

